# Connecticut

**Published:** 1897-03

**Authors:** 


### LEGISLATION.

*Connecticut.* Representative Adrian Wadsworth, of Farmington, and G. E. Manchester, of Winchester, have been appointed a committee by the State Dairymen's Association to confer with the State Board of Health concerning the draft of a bill to be presented at the current session of the Legislature, requiring improved sanitation in stables and barns where milk-cows are kept. Some bill of this character will be urged upon the attention of the General Assembly. Mr. Wadsworth, of the committee, is a member of the present House and a director of the Dairymen's Association. He is a graduate of the Sheffield Scientific School at Yale. and is the Secretary and Treasurer of the Farmington Creamery, Mr. Manchester is also an active and influential farmer in his section of the State. The draft of the bill has not yet been made. It is expected that the State Board of Health will side materially with the Dairymen's Association in the plans that are to be proposed relative to the sanitation of stables and barns. The result of the movement has been to head off the tuberculosis advocates in the attempt that was to be made by them to secure the support of the Health Board. There are members of the State Health Board who are opposed to the course that has been pursued during the past two years in enforcing the tuberculosis laws. For this reason the coalition between the Board and the Dairymen's Association will be the more effective.

Bills have been introduced in the Senate and House repealing the Act of 1895, relating to tuberculosis in cattle. John I. Hutchinson, of Essex, introduced the measure in the House. Senators Brown and Mattoon are the backers of the repealing bill in the Senate.

